# The role of E2F1 in promoting EIF4EBP1 transcription in cryptorchid mice: association with autophagy in germ cells

**DOI:** 10.3389/fgene.2025.1536672

**Published:** 2025-05-23

**Authors:** Jianguo Zhang, Yanhui Liu, Hailong Zhang, Lin Yang, Danjing Sun, Lili Xiao, Xiaoyun Wang, Xiangming Wu

**Affiliations:** Department of Pediatric Surgery, Inner Mongolia Maternal and Child Healthcare Hospital, Hohhot, China

**Keywords:** EIF4EBP1, E2F1, cryptorchidism, apoptosis, autophagy

## Abstract

**Introduction:**

Cryptorchidism can cause excessive germ cell autophagy and apoptosis to impair fertility. This study investigates the role of E2F1 in regulating EIF4EBP1 expression and its contribution to excessive autophagy and apoptosis in cryptorchidism.

**Methods:**

A cryptorchidism mouse model was established through surgical intervention, while an *in vitro* cryptorchid spermatogonial cell model was created using heat stress. Expression levels of EIF4EBP1 and key proteins involved in autophagy and apoptosis were assessed by reverse transcription quantitative polymerase chain reaction (RT-qPCR) and Western blotting Testicular damage and fibrosis were evaluated through HE staining and Masson staining. Serum and testicular testosterone levels were measured, alongside markers of oxidative stress. The regulatory role of E2F1 on EIF4EBP1 was confirmed by dual-luciferase reporter assays and ChIP. Further analysis of the effects of E2F1 and EIF4EBP1 on testicular damage, apoptosis, and autophagy was performed by manipulating EIF4EBP1 expression.

**Results:**

In the cryptorchidism mouse model, reduced testicular volume and weight, increased testicular damage and fibrosis, decreased testosterone levels, and impaired sperm count and vitality were observed. In the *in vitro* cryptorchid spermatogonial cell model, cell viability was reduced, while oxidative stress was elevated. Both autophagy and apoptosis were exacerbated in these models. EIF4EBP1 expression was upregulated, and its knockdown ameliorated the adverse effects. E2F1 was identified as an upstream regulator of EIF4EBP1, with knockdown of E2F1 significantly decreasing EIF4EBP1 promoter activity and binding. Inhibition of E2F1 using HLM006474 reduced EIF4EBP1 expression, while EIF4EBP1 overexpression aggravated autophagy and apoptosis.

**Conclusion:**

E2F1 regulates EIF4EBP1 expression in cryptorchidism, contributing to excessive autophagy and apoptosis. Inhibiting E2F1 reduces these pathological processes, alleviating testicular damage and improving fertility, highlighting potential therapeutic targets for cryptorchidism.

## 1 Introduction

Cryptorchidism is a common congenital disorder of the male urogenital system, affecting 2%–8% of full-term and 10%–20% of preterm male infants (Kubarsepp et al., 2022). Its etiology involves genetic, hormonal, and environmental factors, as well as their complex interactions (Barthold et al., 2016). Cryptorchidism disrupts gonadal development and impairs fertility, with its pathological hallmark being defective spermatogenesis. (Cargnelutti et al., 2022). The abnormal abdominal environment exposes undescended testes to elevated temperatures, causing significant morphological, functional, and gene expression alterations in both germ and somatic cells (Rodprasert et al., 2024; Kato et al., 2022; Thorup et al., 2023). Heat-stressed damage to testicular cells and hormones contributes to spermatogenic failure, with common manifestations including seminiferous tubule atrophy and germ cell apoptosis (Gao et al., 2022; Aldahhan and Stanton, 2021). Although orchidopexy can reposition the testes, residual structural and functional abnormalities often persist, potentially compromising semen quality in adulthood (Liu et al., 2022; Kaselas et al., 2024).

Understanding the molecular mechanisms underlying cryptorchidism is thus critical for elucidating its pathogenesis and developing effective interventions. (Okugi et al., 2022; Fan et al., 2021). Autophagy, a lysosome-mediated degradation process, maintains cellular homeostasis by recycling damaged organelles and macromolecules (Liu et al., 2023). In cryptorchidism, aberrant autophagy contributes to germ cell degradation, impairing spermatogenesis and fertility (Yefimova et al., 2019). Targeting autophagy pathways may thus offer new therapeutic opportunities.

This study identifies EIF4EBP1, a translation inhibitor modulated by mTORC1, as a pivotal regulator of autophagy in cryptorchidism (Aguilar-Valles et al., 2021). Bioinformatics analysis suggests that E2F1, an upstream transcription factor of EIF4EBP1, may drive excessive autophagy under high-temperature stress. While E2F1 is known to regulate cell cycle progression, its role in cryptorchidism remains unexplored (Jing et al., 2022; Jorgez et al., 2021; Polager et al., 2008). Here, we hypothesize that E2F1-induced upregulation of EIF4EBP1 exacerbates germ cell autophagy and apoptosis, contributing to cryptorchidism pathogenesis. This study aims to elucidate the E2F1-EIF4EBP1 axis, providing insights into cryptorchidism and potential therapeutic targets.

## 2 Methods

### 2.1 Bioinformatics analysis

The datasets GSE149084 and GSE25518 related to cryptorchidism were obtained from the GEO database. DEGs in cryptorchidism compared to normal testes were identified with a significance level of *P* < 0.01. Autophagy-related differential expression genes were sourced from the HAMdb database. Protein interaction analysis was conducted using STRING to identify core targets. The hTFtarget system was used to predict upstream transcription factors of EIF4EBP1. The Jvenn system was utilized to generate Venn diagrams showing the intersections among different datasets.

### 2.2 Reagents and instruments

Reagents: Male BALB/c mice (4 weeks old, 12–16 g) were purchased from Hunan Slyke Jinda Experimental Animal Co., Ltd., with production license No.: SCXK (Xiang)2024–0,009; The short hairpin RNA targeting EIF4EBP1 (sh-EIF4EBP1), the EIF4EBP1 overexpression plasmid (oe-EIF4EBP1), and the negative controls for lentiviral infection (including sh-NC and oe-NC) were purchased from Genomeditech, Ltd. (Shanghai, China); GC-1 spg (CL-0600, Procell Life, Wuhan, China); Fetal Bovine Serum (FBS, A5670701, Thermo Fisher Scientific, MA, United States); Penicillin-Streptomycin (10,000 U/mL, 15140122, Thermo Fisher Scientific, MA, United States); Dulbecco’s Modified Eagle Medium (DMEM, 11965092, Thermo Fisher Scientific, MA, United States); Lipofectamine™ 2000 Transfection Reagent (11668027, Thermo Fisher Scientific, MA, United States); HLM006474 (324461, Merck, Darmstadt, Germany); Dimethyl Sulfoxide (DMSO, D2650, Sigma-Aldrich, St. Louis, MO, United States); MTT Solution (HY-15924, Medchemexpress, New Jersey, United States); PBS (AM9624, Thermo Fisher Scientific, MA, United States); Terminal deoxynucleotidyl transferase dUTP nick end labeling (TUNEL) Apoptosis Detection Kit (C1090, Beyotime, Shanghai, China); DCFH-DA (HY-D0940, Medchemexpress, New Jersey, United States); Anti-Fade Mounting Medium (F4680, Sigma-Aldrich, MO, United States); Dual-Luciferase Reporter Assay Kit (RG027, Beyotime, Shanghai, China); Simple ChIP Enzymatic Chromatin IP Kit (9002S, Cell Signaling, Danvers, MA, United States); IgG Antibody (SAB5600195, Sigma-Aldrich, St. Louis, Missouri, United States); Isoflurane (R510-22-16, RWD, Shenzhen, China); Metronidazole (HY-B0318, Medchemexpress, New Jersey, United States); Hematoxylin and Eosin (HE) Staining Kit (C0105S, Beyotime, Shanghai, China); Masson’s Trichrome Staining Kit (C0189S, Beyotime, Shanghai, China); RIPA Lysis Buffer (P0013B, Beyotime, Shanghai, China); BCA Protein Assay Kit (P0010, Beyotime, Shanghai, China); SDS-PAGE (P0531S, Beyotime, Shanghai, China); PVDF Membranes (ab133411, Abcam, Cambridge, UK); Goat Serum (C0265, Beyotime, Shanghai, China); Primary Antibodies: Cleaved Caspase-3 (9661, 1:1000, Cell Signaling Technology, Beverly, MA, United States), LC3 (A5618, 1:500, ABclonal, Wuhan, China), Beclin-1 (ab207612, 1:2000, Abcam, Cambridge, England), P62 (ab314504, 1:1000, Abcam, Cambridge, England), EIF4EBP1 (A24691, 1:500, ABclonal, Wuhan, China), E2F1 (ab314311, 1:1000, Abcam, Cambridge, England), GAPDH (A19056, 1:50000, ABclonal, Wuhan, China); HRP-Conjugated Secondary Antibody (AS014, 1:2000, ABclonal, Wuhan, China); Trizol Reagent (R0016, Beyotime, Shanghai, China); SOD Assay Kit (19160, Sigma-Aldrich, St. Louis, MO, United States); MDA Assay Kit (ab238537, Abcam, Cambridge, UK); GSH/GSSG Ratio Detection Assay Kit (ab138881, Abcam, Cambridge, UK); Testosterone ELISA Kit (PT872, Beyotime, Shanghai, China); RT-qPCR Kit (D7268S, Beyotime, Shanghai, China); Non-fat Milk Powder (P0216, Beyotime, Shanghai, China).

Instruments: ELISA Reader (1410101, Thermo Fisher Scientific, MA, United States); Fluorescence Microscope (DM2500, Leica, Wetzlar, Germany); BeyoECL Star (A38554, Thermo Fisher, Massachusetts, United States); Luciferase Detection System (E1500, Promega, Madison, Wisconsin, United States); ImageJ Software (V1.8.0.112, NIH, Madison, WI, United States); FlowJo (v10.8.2, BD, Ashland, Oregon, United States); GraphPad Prism 9.0 (Dotmatics, Boston, MA, United States).

### 2.3 Animal handling and grouping

Before the experiment, the mice were acclimated for 3 days in a suitable environment (temperature 18°C–26°C, humidity 40%–70% RH, 12-h light/dark cycle, with free access to water and food). This study strictly adhered to the 3 R principles and was approved by the Animal Committee of Hunan Evidence-based Biotechnology Co., Ltd. (Ethics Number: XZ23032).

The mice were randomly divided into 6 groups, with 6 mice in each group: Sham, Surgery, Saline, HLM, HLM + oe-EIF4EBP1, and HLM + oe-NC. The Surgery group mice were anesthetized with 3% isoflurane gas delivered via a mask, and 1% isoflurane gas was maintained during the surgery. The mice were placed on the operating table in a supine position, and a 2 cm incision was made in the midline of the lower abdomen. The abdominal cavity was opened, and the bilateral testes were gently exteriorized and pulled into the abdominal cavity. The testicular pedicles were cut, and the testes were sutured and fixed within the abdominal cavity. In the Sham group, the abdominal cavity was opened, and the bilateral testes were exposed without being cut or fixed, allowing them to remain in their original position (Zheng et al., 2019).

For the lentivirus injection, oe-EIF4EBP1 and oe-NC expressing lentiviruses were injected into the bilateral testes of the mice before suturing (Ikawa et al., 2002). After suturing the abdominal cavity, all mice were administered metronidazole to prevent infection. The E2F1-inhibitor, HLM006474 (12.5 mg/kg) was administered via intraperitoneal injection to the mice three times a week for 6 weeks (Yi et al., 2023). The HLM group received only HLM006474, while the Saline group was injected with an equivalent volume of saline as a negative control. All mice were kept under identical suitable conditions.

At week 6, the mice were euthanized via an intraperitoneal injection of 1% sodium pentobarbital (150 mg/kg). Peripheral blood, testicular tissue, and epididymides were collected. Testicular volume and weight were measured using a precision caliper and electronic balance. The epididymides were dissected to collect sperm, which were then observed under a microscope, and the sperm count and motility ratio (motility ratio = motile sperm count/total sperm count) were recorded using a hemocytometer.

### 2.4 HE staining

Mouse testicular tissues were fixed in 10% neutral buffered formalin, then dehydrated in a graded ethanol series and cleared with xylene. The tissues were embedded in paraffin and sectioned. The paraffin sections were dewaxed in xylene and rehydrated through an alcohol-to-water gradient. The sections were stained with an HE staining kit, cleared again in xylene, and dehydrated through graded ethanol. Finally, the sections were observed and photographed using a standard optical microscope.

### 2.5 Masson’s trichrome staining

Deparaffinize mouse testicular tissue sections to water. Stain with hematoxylin for 5 min, followed by staining with Biebrich Scarlet-Acid Fuchsin solution for 10 min. Then, treat with phosphomolybdic acid solution for 2 min, and finally stain with light green solution for 1 min. After rinsing with 1% acetic acid solution, dehydrate the sections using a graded series of ethanol, clear with xylene, and observe under a standard light microscope for photography.

### 2.6 Cell culture and treatment

The GC-1 spg mouse spermatogonial cells were cultured in DMEM medium with 10% FBS, 100 U/mL penicillin, and 100 μg/mL streptomycin in a humidified incubator at 37°C with 5% CO2. To create a cryptorchidism model, cells were exposed to 42°C for 2 h (Shahat et al., 2020). For HLM006474 treatment, cells were incubated with 40 μM HLM006474 dissolved in DMSO for 24 h (Ma et al., 2008), with an equal volume of DMSO added to the medium as a negative control.

### 2.7 Construction of cells with stable gene transfection

The mouse spermatogonial cell line GC-1 spg was seeded into culture plates, and lentiviral infection was performed when the cells reached 60% confluence. After 48 h of infection, stable infected cells were selected by adding the appropriate antibiotics, and the successful alteration of gene expression was confirmed by reverse transcription quantitative polymerase chain reaction (RT-qPCR) and Western blotting (WB) analysis.

### 2.8 MTT

GC-1 spg cells in the logarithmic growth phase were plated into 96-well plates at a density of 5,000 cells per well, with five replicates per group. Then, 10 μL of MTT solution was added to each well. The plates were incubated at 37°C for 4 h. Absorbance at 562 nm was measured using a microplate reader.

### 2.9 Biochemical analysis

Cell culture supernatants, mouse serum, and testicular tissue homogenates were collected, and the levels of SOD, MDA, GSH/GSSG, and testosterone in the samples were measured using commercial assay kits. The procedures were performed according to the instructions provided with each kit. Absorbance measurements were taken using a microplate reader.

### 2.10 Detection of reactive oxygen species (ROS)

Add 10 μM DCFH-DA to each group of GC-1 spg cells and incubate in the dark for 30 min in a cell culture incubator. Remove the unreacted probe, and then mount the slides using an anti-fade mounting medium. Observe and capture images using a fluorescence microscope. Fluorescence intensity was evaluated using ImageJ software.

### 2.11 TUNEL

Fix each group of GC-1 spg cells with 4% paraformaldehyde for 30 min, followed by incubation with PBS containing 0.1% Triton X-100 at room temperature for 5 min. Prepare the TUNEL detection solution according to the instructions provided with the TUNEL assay kit, and add 50 μL of the solution to each sample. Incubate in the dark at 37°C for 60 min. Add DAPI working solution (1 μg/mL) and incubate in the dark at room temperature for 30 min. After mounting the slides with an anti-fade mounting medium, observe the samples under a fluorescence microscope. The apoptosis rate was calculated using ImageJ software.

### 2.12 Dual-luciferase reporter assay

The mouse EIF4EBP1 promoter region DNA sequence was retrieved from the UCSC database (http://genome.ucsc.edu/), followed by prediction of E2F1 binding sites in the EIF4EBP1 promoter region using JASPAR (https://jaspar.elixir.no/). Mutations at the top three binding sites were performed. Subsequently, the amplified wild-type (WT) and mutated (MUT) EIF4EBP1 promoter sequences were inserted into the pGL3-Basic luciferase reporter vectors, which were transfected with sh-NC and sh-E2F1 plasmids into GC-1 spg cells using Lipofectamine 2000 and incubated at 37°C with 5% CO_2_ for 48 h. The luciferase activity was measured using a luciferase reporter assay kit.

### 2.13 Chromatin immunoprecipitation (ChIP) assay

According to the Simple ChIP Enzymatic Chromatin IP Kit instructions, treat GC-1 spg cells with formaldehyde for 15 min. Quench the protein-DNA crosslinking with glycine. Digest the chromatin with micrococcal nuclease and resuspend in ChIP buffer. Perform the immunoprecipitation (IP) reaction by adding E2F1 antibody (1:30) or IgG and incubate overnight at 4°C. Elute the chromatin and reverse the crosslinks, then purify the DNA for qPCR analysis.

### 2.14 WB

Obtain total protein lysates from cell and testicular tissue homogenates using RIPA lysis buffer. Measure protein concentration using a BCA protein assay kit, then load the protein samples onto an SDS-PAGE gel for electrophoresis. Transfer the samples to PVDF membranes, block with 5% non-fat milk, and incubate overnight at 4°C with primary antibodies against Cleaved Caspase-3, LC3, Beclin-1, P62, E2F1, EIF4EBP1, and GAPDH. Subsequently, incubate with HRP-conjugated secondary antibodies at room temperature for 2 h. Develop the blots using BeyoECL Star and analyze band density with ImageJ software, using GAPDH as an internal control for quantification.

### 2.15 RT-qPCR

According to the manufacturer’s instructions for the Trizol reagent kit, extract total RNA from cell and testicular tissue homogenates. Use RT-qPCR kits and specific primers (see Table 1) to set up reactions for E2F1 and EIF4EBP1. Calculate relative expression levels using the 2^−ΔΔCt^ formula, with GAPDH as the internal control.

**TABLE 1:** Primer sequences.

*Gene*	*Direction*	*Sequence (5′-3′)*	*Product (bp)*	*Tm (°C)*
*E2F1*	F	*GGA​TCT​GGA​GAC​TGA​CCA​TCA​G*	115	60
	R	*GGT​TTC​ATA​GCG​TGA​CTT​CTC​CC*		
*EIF4EBP1*	F	*GGA​GAG​CTG​CAC​AGC​ATT​CAG​G*	126	60
	R	*GGA​GGT​ATG​TGC​TGG​TGT​TCA​C*		
*GAPDH*	F	*CAT​CAC​TGC​CAC​CCA​GAA​GAC​TG*	153	60
	R	*ATG​CCA​GTG​AGC​TTC​CCG​TTC​AG*		

### 2.16 Statistical analysis

Statistical analyses were conducted using Prism 9 software, with data presented as mean ± SD. T-tests were applied for comparisons between two groups, while one-way or two-way ANOVA followed by Tukey’s *post hoc* test was used for comparisons among three or more groups. A P-value of <0.05 was considered statistically significant.

## 3 Results

### 3.1 EIF4EBP1 is highly expressed in cryptorchidism mouse models

**FIGURE 1:**
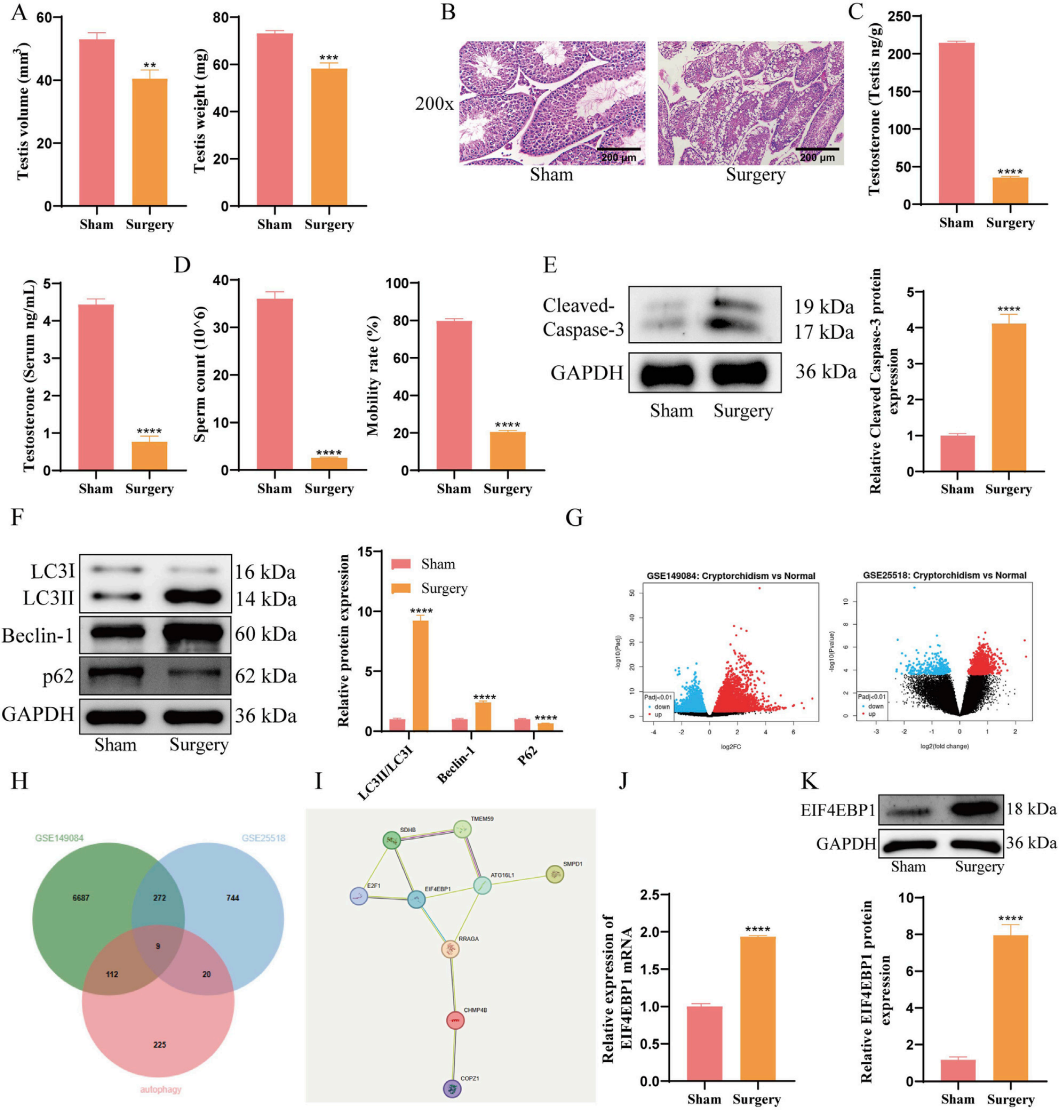
EIF4EBP1 is Highly Expressed in Cryptorchidism Mouse Models. **(A)** Weight and volume of mouse testicular tissue, data analyzed using t-test. **(B)** Evaluation of testicular tissue damage using HE staining (200×, Scale = 200 μm). **(C)** Measurement of testosterone levels in serum and testicular tissue using assay kits, data analyzed using t-test. **(D)** Assessment of sperm motility under a microscope and calculation of sperm count, data analyzed using t-test. **(E)** WB analysis of Cleaved Caspase-3, data analyzed using t-test. **(F)** WB analysis of LC3, Beclin-1, and p62 expression levels in testicular tissue, data analyzed using Two-way ANOVA, and the *post hoc* test was conducted using Tukey’s. **(G)** Identification of differentially expressed genes (DEGs) between Normal and Cryptorchidism in the GSE149084 and GSE25518 datasets. **(H)** The intersection analysis of DEGs from GSE149084 and GSE25518, along with autophagy-related genes, was performed using jvenn. **(I)** PPI analysis of intersecting factors, including SDHB, TMEM59, E2F1, EIF4EBP1, ATG15L1, SMPD1, RRAGA, CHMP4B, and COPZ1, was conducted using STRING. **(J)** Detection of EIF4EBP1 expression in testicular tissue by RT-qPCR, data analyzed using t-test. **(K)** Detection of EIF4EBP1 expression by WB, data analyzed using t-test. **P < 0.01; ***P < 0.001; ****P < 0.0001.

A cryptorchidism mouse model was established surgically to investigate the pathological mechanisms of the condition. Results demonstrated a significant reduction in testicular volume and weight in the Surgery group compared to the Sham group (Figure 1A). HE staining revealed pronounced testicular injury in the Surgery group (Figure 1B). Additionally, testosterone levels in both serum and testis were markedly lower in the Surgery group, along with reduced sperm count and motility (Figures 1C,D), which confirmed the successful establishment of the cryptorchidism model.

The results of WB analysis showed that the Surgery group exhibited significantly increased levels of Cleaved Caspase-3, LC3II/LC3I, and Beclin-1, while P62 were downregulated (Figures 1E,F). These findings indicated enhanced autophagy and apoptosis in the cryptorchidism model. To explore the mechanism underlying excessive autophagy in germ cells, we used jvenn (https://jvenn.toulouse.inrae.fr/app/example.html) to perform a cross-analysis of the differentially expressed genes (DEGs) from GSE149084 and GSE25518 (Figure 1G) alongside autophagy-related genes. This analysis identified nine overlapping factors: SDHB, TMEM59, E2F1, EIF4EBP1, ATG15L1, SMPD1, RRAGA, CHMP4B, and COPZ1 (Figure 1H). Subsequently, we conducted a protein-protein interaction (PPI) analysis of these nine overlapping factors using STRING (https://cn.string-db.org/cgi/input?sessionId=bhaAMvIiNIFZ&input_page_active_form=multiple_identifiers) (Szklarczyk et al., 2023). The results revealed that EIF4EBP1 was a key gene among the DEGs related to cryptorchidism and autophagy (Figure 1I) (Bardou et al., 2014). Subsequent RT-qPCR and WB analyses revealed that EIF4EBP1 expression was significantly upregulated in the testicular tissues of cryptorchidism model mice (Figures 1J,K).

### 3.2 EIF4EBP1 is highly expressed in germ cells of cryptorchidism

**FIGURE 2:**
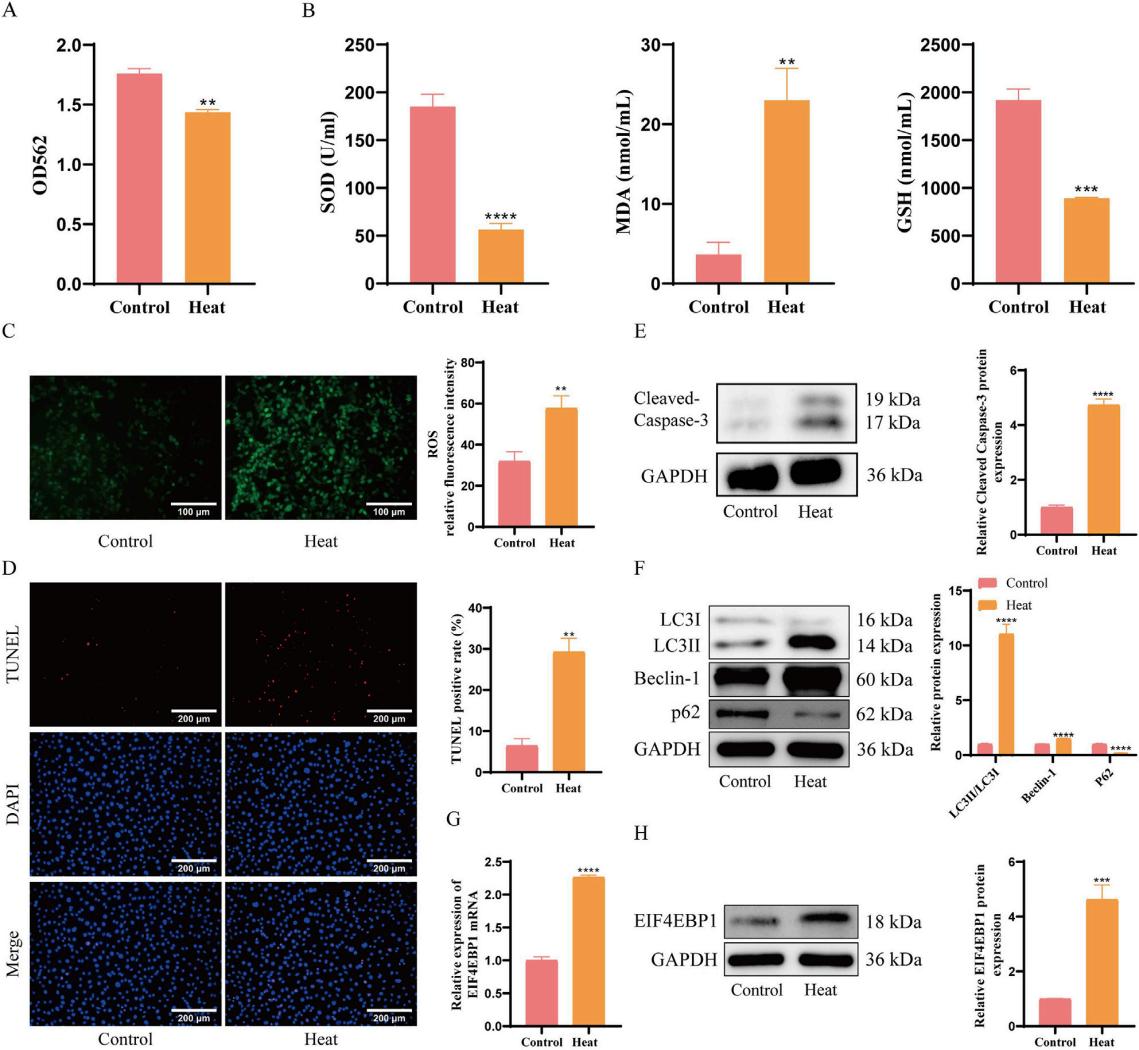
EIF4EBP1 is Highly Expressed in Germ Cells of Cryptorchidism. **(A)** Cell viability assessment using the MTT assay, data analyzed using t-test. **(B)** Measurement of oxidative stress markers (SOD, MDA, and GSH) using ELISA, data analyzed using t-test. **(C)** Detection of ROS levels through fluorescence staining (400×, Scale = 100 μm), data analyzed using t-test. **(D)** Detection of cell apoptosis using TUNEL assay (200×, Scale = 200 μm), data analyzed using t-test. **(E)** WB analysis of Cleaved Caspase-3, data analyzed using t-test. **(F)** WB analysis of LC3, Beclin-1, and p62 expression levels, data analyzed using Two-way ANOVA, and the *post hoc* test was conducted using Tukey’s. **(G)** Analysis of EIF4EBP1 expression using RT-qPCR, data analyzed using t-test. **(H)** Detection of EIF4EBP1 expression using WB, data analyzed using t-test. **P < 0.01; ***P < 0.001; ****P < 0.0001.

The expression of EIF4EBP1 in cryptorchidism was further investigated using *in vitro* experiments. A cryptorchid spermatogonial cell model was established under heat stress conditions. Results from the MTT assay revealed significantly reduced cell viability in the Heat group compared to the Control group (Figure 2A). Biochemical analyses and fluorescence staining showed that oxidative stress markers, such as SOD and GSH/GSSG, were significantly reduced, while MDA and ROS levels were markedly elevated in the Heat group (Figures 2B,C), confirming successful model establishment.

TUNEL staining and WB analyses results further demonstrated that the percentage of apoptotic cells was significantly higher in the Heat group (Figure 2D). Consistently, the expression of apoptosis-related protein Cleaved Caspase-3 and autophagy-related proteins LC3II/LC3I and Beclin-1 were upregulated, while autophagy-related protein P62 were significantly downregulated (Figures 2E,F). Furthermore, RT-qPCR and WB demonstrated a significant increase in EIF4EBP1 expression in the Heat group compared to the Control group (Figures 2G,H). These findings suggest that the upregulation of EIF4EBP1 was associated with enhanced signs of apoptosis and autophagy in germ cells, providing insight into its potential role in cryptorchidism pathology.

### 3.3 EIF4EBP1 knockdown reduces apoptosis and excessive autophagy in germ cells affected by cryptorchidism

**FIGURE 3:**
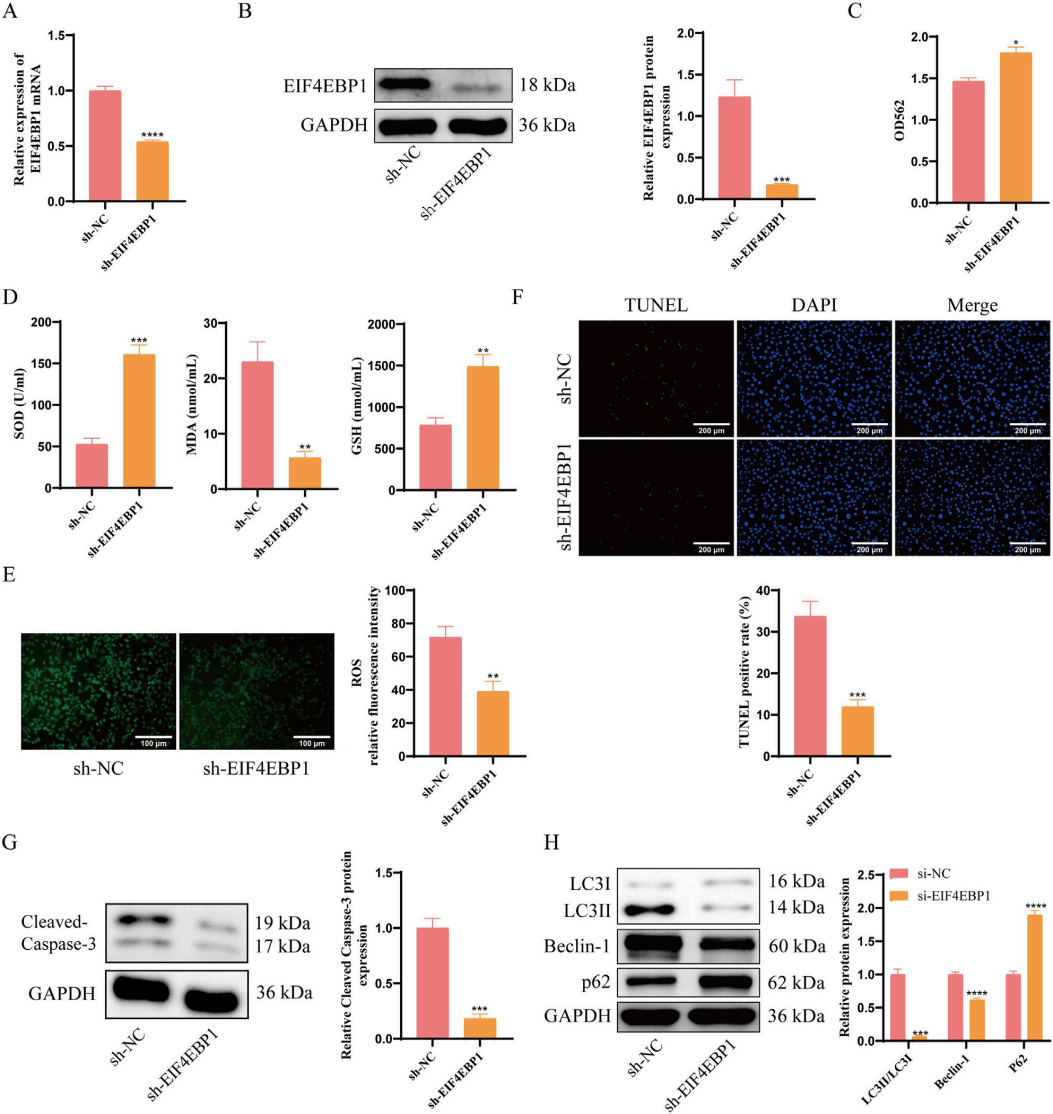
EIF4EBP1 knockdown reduces apoptosis and excessive autophagy in germ cells affected by cryptorchidism. **(A)** Detection of EIF4EBP1 expression using RT-qPCR, data analyzed using t-test. **(B)** Analysis of EIF4EBP1 expression using WB, data analyzed using t-test. **(C)** Assessment of cell viability using the MTT assay, data analyzed using t-test. **(D)** Measurement of oxidative stress markers (SOD, MDA, and GSH) using ELISA, data analyzed using t-test. **(E)** Detection of ROS levels via fluorescence staining, data analyzed using t-test. **(F)** Detection of cell apoptosis using the TUNEL assay, data analyzed using t-test. **(G)** WB analysis of Cleaved Caspase-3, data analyzed using t-test. **(H)** WB analysis of LC3, Beclin-1, and P62 expression levels, data analyzed using Two-way ANOVA, and the *post hoc* test was conducted using Tukey’s. *P < 0.05; **P < 0.01; ***P < 0.001; ****P < 0.0001.

GC-1 spg cells were transduced with a recombinant lentivirus expressing sh-EIF4EBP1. This established an EIF4EBP1 knockdown model within the cryptorchid spermatogonial cell model. RT-qPCR and WB analyses confirmed a significant reduction in EIF4EBP1 expression in the sh-EIF4EBP1 group compared to the sh-NC group (Figures 3A,B). Following EIF4EBP1 knockdown, cell viability in the cryptorchid spermatogonial cell model significantly improved (Figure 3C), accompanied by reductions in ROS and MDA (Figures 3D,E).

Assessment of apoptosis and autophagy revealed that EIF4EBP1 knockdown significantly reduced the percentage of apoptotic cells (Figure 3F). Notably, the expression levels of apoptosis-related proteins Cleaved Caspase-3 and autophagy-related proteins LC3II/LC3I and Beclin-1 were decreased, whereas autophagy-related protein P62 was significantly increased in the sh-EIF4EBP1 group (Figures 3G,H). Taken above, EIF4EBP1 knockdown reduces signs of autophagy and apoptosis in germ cells exposed to cryptorchid conditions. The results highlight EIF4EBP1 as a potential therapeutic target for managing cryptorchidism-associated germ cell damage.

### 3.4 E2F1 is an upstream transcription factor of EIF4EBP1

**FIGURE 4:**
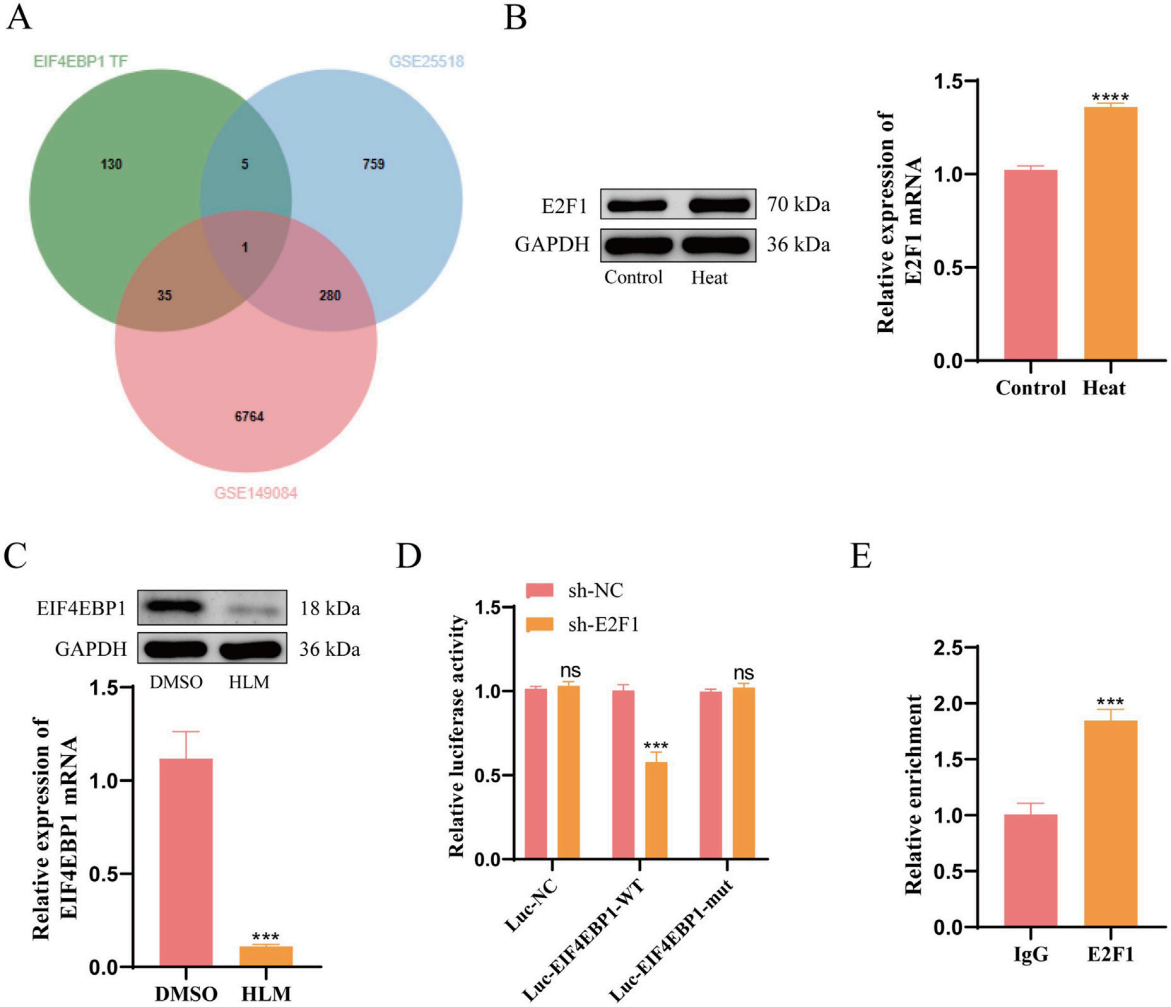
E2F1 is an Upstream Transcription Factor of EIF4EBP1. **(A)** Intersection analysis of EIF4EBP1 transcription factors (TFs) with data from GSE149084 and GSE25518. **(B)** WB analysis of E2F1 expression, data analyzed using t-test. **(C)** WB analysis of EIF4EBP1 expression, data analyzed using t-test. **(D)** Dual-luciferase reporter assay to evaluate the effect of E2F1 on EIF4EBP1 promoter activity, data analyzed using Two-way ANOVA, and the *post hoc* test was conducted using Tukey’s. **(E)** Chromatin immunoprecipitation (ChIP) assay to determine the enrichment of the EIF4EBP1 promoter in E2F1, data analyzed using t-test. ^ns^ *P*>0.05; ***P < 0.001; ****P < 0.0001.

To further investigate the mechanisms underlying excessive autophagy in germ cells during cryptorchidism, bioinformatics analysis identified E2F1 as an upstream transcription factor of EIF4EBP1, intersecting with differentially expressed genes associated with both cryptorchidism and autophagy (Figure 4A). WB analysis of E2F1 expression in cryptorchidism spermatogonial cells revealed significant upregulation in the Heat group compared to the Control group (Figure 4B). Moreover, treatment with an E2F1-inhibitor significantly decreased EIF4EBP1 expression in the cryptorchid spermatogonial cell model (Figure 4C).

To elucidate the regulatory relationship between E2F1 and EIF4EBP1 at the transcriptional level, we conducted dual-luciferase reporter assays and ChIP experiments. The dual-luciferase reporter assay demonstrated that E2F1 knockdown reduced the activity of the wild-type EIF4EBP1 promoter, but had no effect on the mutated version (Figure 4D). Furthermore, ChIP analysis revealed significantly greater enrichment of E2F1 at the EIF4EBP1 promoter region compared to the IgG control group (Figure 4E). These findings confirm that E2F1 functions as an upstream transcription factor for EIF4EBP1.

### 3.5 Inhibition of E2F1 relieves apoptosis and excessive autophagy in germ cells of cryptorchidism through EIF4EBP1

**FIGURE 5:**
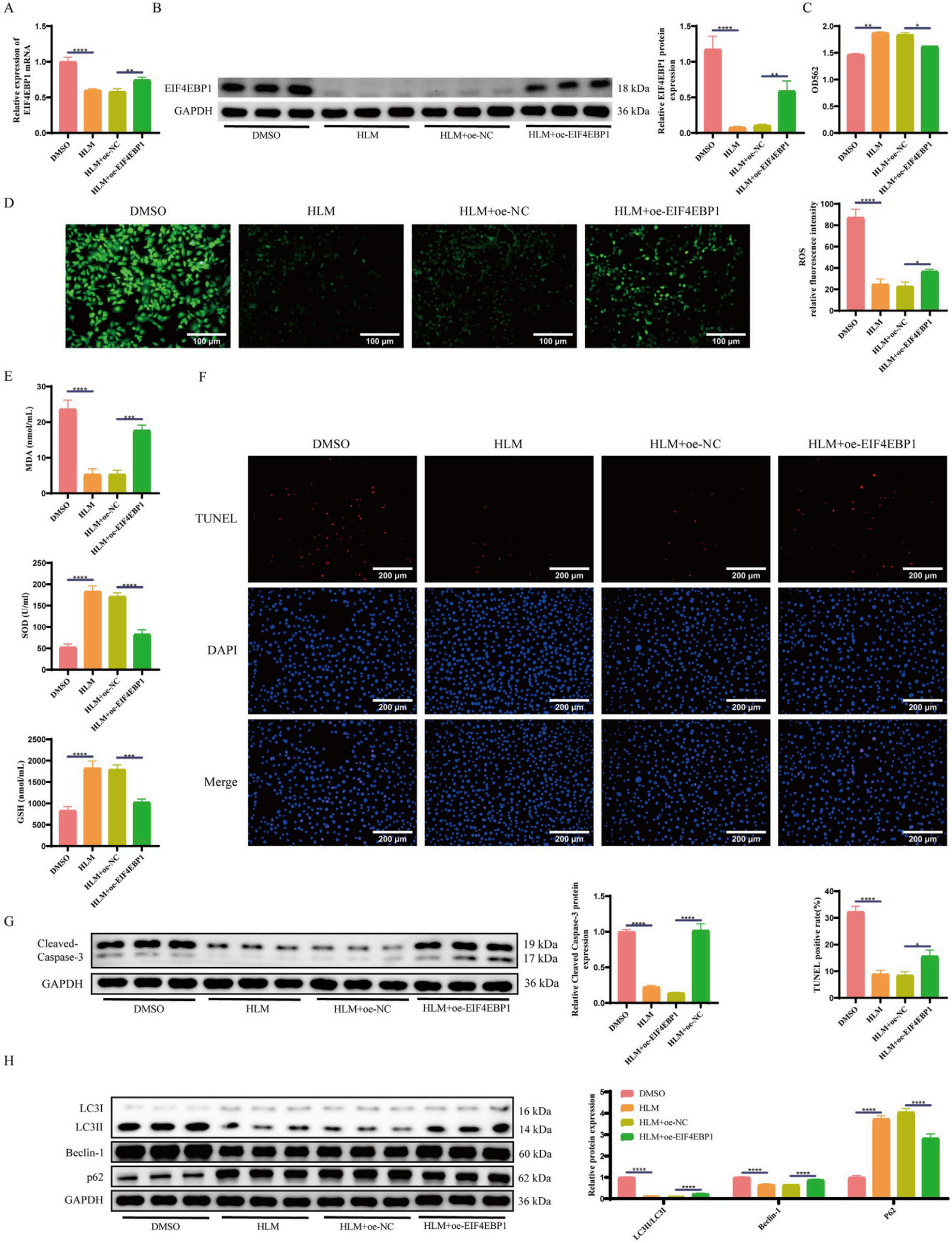
Inhibition of E2F1 Relieves Apoptosis and Excessive Autophagy in Germ Cells of Cryptorchidism through EIF4EBP1. **(A)** Detection of EIF4EBP1 expression using RT-qPCR, data analyzed using one-way ANOVA, and the *post hoc* test was conducted using Tukey’s. **(B)** Analysis of EIF4EBP1 expression using WB, data analyzed using one-way ANOVA, and the *post hoc* test was conducted using Tukey’s. **(C)** Assessment of cell viability using the MTT assay, data analyzed using one-way ANOVA, and the *post hoc* test was conducted using Tukey’s. **(D)** Detection of ROS levels via fluorescence staining (400×, Scale = 100 μm), data analyzed using one-way ANOVA, and the *post hoc* test was conducted using Tukey’s. **(E)** Measurement of oxidative stress markers (SOD, MDA, and GSH) using ELISA, data analyzed using one-way ANOVA, and the *post hoc* test was conducted using Tukey’s. **(F)** Detection of cell apoptosis using the TUNEL assay (200×, Scale = 200 μm), data analyzed using one-way ANOVA, and the *post hoc* test was conducted using Tukey’s. **(G)** WB analysis of Cleaved Caspase-3, data analyzed using one-way ANOVA, and the *post hoc* test was conducted using Tukey’s. **(H)** WB analysis of LC3, Beclin-1, and p62 expression levels, data analyzed using Two-way ANOVA, and the *post hoc* test was conducted using Tukey’s. *P < 0.05; **P < 0.01; ***P < 0.001; ****P < 0.0001.

To further confirm whether E2F1 influences apoptosis and autophagy in cryptorchidism germ cells via EIF4EBP1, we generated GC-1 spg cells overexpressing EIF4EBP1 and established a cryptorchid spermatogonial cell model. These cells were treated with the E2F1-inhibitor, HLM006474. RT-qPCR and WB analyses revealed that EIF4EBP1 expression was significantly lower in the HLM006474-treated group compared to the DMSO control group (Figures 5A,B). Moreover, the overexpression of EIF4EBP1 restored its levels, confirming successful infection.

E2F1 inhibition significantly improved cell viability and reduced oxidative stress in spermatogonial cells compared to the DMSO group. However, when EIF4EBP1 was overexpressed, cell viability decreased again, and oxidative stress levels increased (Figures 5C–E).

Next, we assessed apoptosis and autophagy. The results indicated that inhibition of E2F1 significantly reduced the proportion of apoptotic cells (Figure 5F) and increased the levels of the apoptosis-related protein Cleaved Caspase-3 and autophagy-related proteins LC3II/LC3I and Beclin-1, while decreasing the levels of the autophagy-related protein P62 (Figures 5G,H). Conversely, EIF4EBP1 overexpression exacerbated both signs of apoptosis and autophagy.

### 3.6 Inhibition of E2F1 Protects testes from damage in cryptorchidism mouse models through EIF4EBP1

**FIGURE 6:**
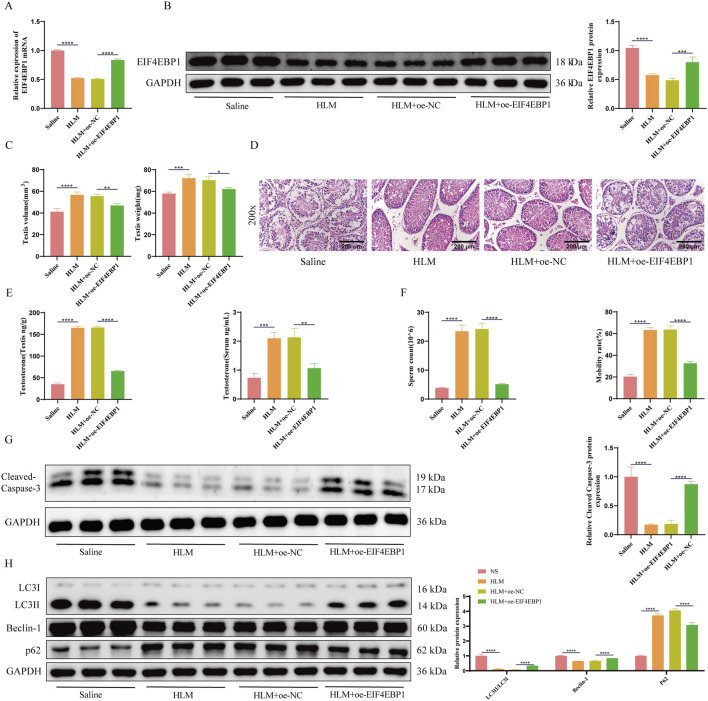
Inhibition of E2F1 Protects Testes from Damage in Cryptorchidism Mouse Models through EIF4EBP1. **(A)** Detection of EIF4EBP1 expression using RT-qPCR, data analyzed using one-way ANOVA, and the *post hoc* test was conducted using Tukey’s. **(B)** Analysis of EIF4EBP1 expression using WB, data analyzed using one-way ANOVA, and the *post hoc* test was conducted using Tukey’s. **(C)** Measurement of testicular tissue weight and volume, data analyzed using one-way ANOVA, and the *post hoc* test was conducted using Tukey’s. **(D)** Assessment of testicular tissue damage using HE staining (200×, Scale = 200 μm). **(E)** Determination of testosterone levels in serum and testicular tissue using assay kits, data analyzed using one-way ANOVA, and the *post hoc* test was conducted using Tukey’s. **(F)** Evaluation of sperm motility and sperm count under a microscope, data analyzed using one-way ANOVA, and the *post hoc* test was conducted using Tukey’s. **(G)** WB analysis of Cleaved Caspase-3, data analyzed using one-way ANOVA, and the *post hoc* test was conducted using Tukey’. **(H)** WB analysis of LC3, Beclin-1, and p62 expression levels, data analyzed using Two-way ANOVA, and the *post hoc* test was conducted using Tukey’s. *P < 0.05; **P < 0.01; ***P < 0.001; ****P < 0.0001.

To investigate whether E2F1 affects testicular damage and fertility in the cryptorchidism mouse model through EIF4EBP1, we injected oe-EIF4EBP1-expressing recombinant lentivirus into the testes of cryptorchidism mice and treated them with the E2F1-inhibitor HLM006474. Successful lentiviral infection was confirmed by fluorescence microscopy (Supplementary Materials). RT-qPCR and WB analyses confirmed that EIF4EBP1 expression was significantly lower in the HLM006474-treated group compared to the saline control group. Overexpression of EIF4EBP1 reversed this decrease, validating successful infection (Figures 6A,B).

Measurements of testicular volume and weight indicated that E2F1 inhibition significantly increased both parameters, whereas EIF4EBP1 overexpression reduced them again (Figure 6C). HE staining revealed that E2F1 inhibition improved testicular damage, while EIF4EBP1 overexpression exacerbated the damage further (Figure 6D).

Biochemical analyses showed that E2F1 inhibition significantly increased testosterone levels in both the serum and testes, but overexpression of EIF4EBP1 reversed this effect (Figure 6E). Additionally, E2F1 inhibition resulted in significant improvements in sperm count and motility, which were diminished upon EIF4EBP1 overexpression (Figure 6F). WB results confirmed that E2F1 inhibition significantly reduced apoptosis and autophagy in the testes, yet EIF4EBP1 overexpression promoted apoptosis and autophagy (Figures 6G,H).

In conclusion, E2F1 inhibition protects the testes from damage in a cryptorchidism mouse model by reducing EIF4EBP1 transcription.

## 4 Discussion

Cryptorchidism is a common reproductive disorder characterized by testicular underdevelopment, spermatogenic dysfunction, and elevated levels of germ cell apoptosis and autophagy (Elamo et al., 2022). In this study, we explored the pivotal role of EIF4EBP1 in the pathophysiology of cryptorchidism and examined the regulatory mechanism of its upstream regulator, E2F1. By conducting both *in vivo* and *in vitro* experiments, we confirmed the association between EIF4EBP1 expression and increased germ cell apoptosis and autophagy in cryptorchidism. These findings offer valuable insights into the molecular mechanisms underlying cryptorchidism and suggest potential therapeutic targets.

Cryptorchidism refers to the failure of the testicles to descend into the scrotum, resulting in their retention along the descent pathway (Lee and Houk, 2013). The scrotum provides an optimal temperature for spermatogenesis, which is lower than the normal body temperature (Rew et al., 2021). The higher temperature in the abdominal cavity can negatively affect sperm production and maturation. Additionally, the absence of testicles from the scrotum impairs its development, leading to reduced fertility (Elamo et al., 2022). Cryptorchidism models are typically created using surgical fixation of the testicles in the abdominal cavity or through heat stress. In our study, we established a cryptorchidism mouse model by surgically fixing the testes in the abdominal cavity, followed by heat stress to generate a cryptorchid spermatogonial cell model. Our results demonstrated that the mice in the Surgery group exhibited significantly reduced testicular volume and weight, with increased damage, fibrosis, reduced testosterone levels, and diminished sperm quantity and activity. In the *in vitro* experiments, heat-stressed spermatogonial cells showed decreased viability and exacerbated oxidative stress responses, confirming successful model establishment. Furthermore, apoptosis and autophagy were significantly increased in the cryptorchidism models.

Apoptosis and autophagy are crucial mechanisms for maintaining the survival and function of reproductive cells. However, in cryptorchidism, these processes often become dysregulated, leading to excessive apoptosis and autophagy in reproductive cells, thus impairing spermatogenesis (Zheng et al., 2019). In this study, we analyzed the state of apoptosis and autophagy in the cryptorchidism models and found that both processes were significantly elevated. To further investigate the molecular pathology, we used bioinformatics analysis to identify EIF4EBP1, a gene differentially expressed in cryptorchidism and autophagy. EIF4EBP1 encodes a protein that regulates protein synthesis and cell growth. RT-qPCR and WB analyses revealed a significant upregulation of EIF4EBP1 in both *in vivo* and *in vitro* cryptorchidism models, suggesting its role in the pathological development of cryptorchidism. Previous studies have shown that EIF4EBP1 is activated under various stress conditions, contributing to increased apoptosis and autophagy by inhibiting protein synthesis (Yang et al., 2021). However, the role of EIF4EBP1 in excessive autophagy in cryptorchidism remained unclear. To address this, we knocked down EIF4EBP1 in the cryptorchid spermatogonial cell model and observed its effects on cell apoptosis and autophagy. Our findings demonstrated that knocking down EIF4EBP1 significantly enhanced cell viability and reduced oxidative stress levels. Moreover, expression levels of apoptosis-related protein Cleaved Caspase-3, as well as autophagy-related proteins LC3II/LC3I and Beclin-1, were markedly reduced, supporting the notion that EIF4EBP1 upregulation is linked to excessive apoptosis and autophagy in reproductive cells. Notably, the increased expression of P62, an autophagy receptor, was observed following EIF4EBP1 knockdown, reflecting reduced autophagic activity and further emphasizing the importance of EIF4EBP1 in regulating autophagy (Kumar et al., 2022). In summary, EIF4EBP1 significantly modulates apoptosis and autophagy in reproductive cells during cryptorchidism, and its increased expression likely contributes to the disease’s progression.

To explore the regulatory mechanisms of EIF4EBP1 in cryptorchidism, we investigated its upstream regulatory factors. Bioinformatics analysis revealed E2F1 as a potential transcription factor upstream of EIF4EBP1. E2F1 is a well-established regulator of the cell cycle, influencing key processes such as cell proliferation, apoptosis, and autophagy. Previous studies have suggested that both overexpression and deletion of E2F1 are implicated in the development of cryptorchidism (Jorgez et al., 2015). Our WB analysis showed that E2F1 was significantly upregulated in the cryptorchid spermatogonial cell model, and inhibition of E2F1 led to a notable decrease in EIF4EBP1 expression. To confirm the transcriptional regulation of EIF4EBP1 by E2F1, we performed dual-luciferase reporter assays and ChIP experiments. The results demonstrated that E2F1 knockdown significantly reduced the transcriptional activity of the EIF4EBP1 promoter, and ChIP assays confirmed the direct interaction of E2F1 with the EIF4EBP1 promoter region. These findings underscore the critical role of E2F1 in regulating EIF4EBP1 expression.

To further validate E2F1’s role in cryptorchidism, we inhibited E2F1 in the cryptorchid spermatogonial cell model and assessed its impact on apoptosis and autophagy. The results showed that E2F1 inhibition significantly reduced EIF4EBP1 expression, improved germ cell viability, and reduced oxidative stress levels. Additionally, the expression of apoptosis- and autophagy-related proteins was substantially suppressed. Importantly, overexpression of EIF4EBP1 reversed the protective effects of E2F1 inhibition, resulting in decreased cell viability and increased levels of apoptosis and autophagy. This further validates the essential role of E2F1 in regulating apoptosis and autophagy in cryptorchidism germ cells via EIF4EBP1. *In vivo*, our studies also demonstrated that E2F1 inhibition mediated by EIF4EBP1 provides a protective effect on the testes in cryptorchidism mice.

In summary, this study is the first to elucidate the critical role of EIF4EBP1 in the pathology of cryptorchidism and identify E2F1 as its upstream regulator. Our research demonstrates that EIF4EBP1 upregulation promotes apoptosis and autophagy in cryptorchidism germ cells, while E2F1 inhibition alleviates these pathological symptoms by downregulating EIF4EBP1 expression. Through a combination of *in vivo* and *in vitro* experiments, we further emphasize the significance of the E2F1-EIF4EBP1 axis in cryptorchidism. These findings not only provide insights into the molecular mechanisms of cryptorchidism but also highlight potential targets for future therapeutic interventions. Further studies should explore the specific molecular pathways of the E2F1-EIF4EBP1 axis in cryptorchidism and investigate how this pathway can be targeted through drug or gene therapy to alleviate the condition. Additionally, the roles of E2F1 and EIF4EBP1 in other reproductive disorders should be explored to better understand the broader implications of this signaling pathway for reproductive health.

## 5 Conclusion

E2F1 is essential in the progression of cryptorchidism through its regulation of EIF4EBP1 expression. Inhibition of E2F1 can effectively reduce apoptosis and excessive autophagy in germ cells of cryptorchidism by downregulating EIF4EBP1 expression, thereby alleviating testicular damage and the decline in fertility in cryptorchidism model mice. This offers a scientific foundation for comprehending the pathology of cryptorchidism and identifying novel therapeutic targets.

## Data Availability

The original contributions presented in the study are included in the article/Supplementary Material, further inquiries can be directed to the corresponding authors.

## References

[B1] Aguilar-Valles A. De Gregorio D. Matta-Camacho E. Eslamizade M. J. Khlaifia A. Skaleka A. (2021). Antidepressant actions of ketamine engage cell-specific translation via eIF4E. Nature 590, 315–319. 10.1038/s41586-020-03047-0 33328636

[B2] Aldahhan R. A. Stanton P. G. (2021). Heat stress response of somatic cells in the testis. Mol. Cell Endocrinol. 527, 111216. 10.1016/j.mce.2021.111216 33639219

[B32] Bardou P. Mariette J. Escudié F. Djemie C. Klopp C. (2014). Jvenn: An interactive venn diagram viewer. BMC Bioinformatics. 15 (1), 293. 10.1186/1471-2105-15-293 PMC4261873 25176396

[B3] Barthold J. S. Reinhardt S. Thorup J. (2016). Genetic, maternal, and environmental risk factors for cryptorchidism: an update. Eur. J. Pediatr. Surg. 26, 399–408. 10.1055/s-0036-1592416 27642851

[B4] Cargnelutti F. Di Nisio A. Pallotti F. Spaziani M. Tarsitano M. G. Paoli D. (2022). Risk factors on testicular function in adolescents. J. Endocrinol. Invest 45, 1625–1639. 10.1007/s40618-022-01769-8 35286610 PMC9360118

[B5] Elamo H. P. Virtanen H. E. Toppari J. (2022). Genetics of cryptorchidism and testicular regression. Best. Pract. Res. Clin. Endocrinol. Metab. 36, 101619. 10.1016/j.beem.2022.101619 35193821

[B6] Fan X. Liu Y. Yue M. Yue W. Ren G. Zhang J. (2021). Effect of cryptorchidism on the histomorphometry, proliferation, apoptosis, and autophagy in boar testes. Anim. (Basel) 11, 1379. 10.3390/ani11051379 PMC8152062 34066291

[B7] Gao Y. Wang C. Wang K. He C. Hu K. Liang M. (2022). The effects and molecular mechanism of heat stress on spermatogenesis and the mitigation measures. Syst. Biol. Reprod. Med. 68, 331–347. 10.1080/19396368.2022.2074325 35722894

[B8] Ikawa M. Tergaonkar V. Ogura A. Ogonuki N. Inoue K. Verma I. M. (2002). Restoration of spermatogenesis by lentiviral gene transfer: offspring from infertile mice. Proc. Natl. Acad. Sci. U. S. A. 99, 7524–7529. 10.1073/pnas.072207299 12032316 PMC124271

[B9] Jing Z. Liu Q. He X. Jia Z. Xu Z. Yang B. (2022). NCAPD3 enhances Warburg effect through c-myc and E2F1 and promotes the occurrence and progression of colorectal cancer. J. Exp. Clin. Cancer Res. 41, 198. 10.1186/s13046-022-02412-3 35689245 PMC9188166

[B10] Jorgez C. J. Seth A. Wilken N. Bournat J. C. Chen C. H. Lamb D. J. (2021). E2F1 regulates testicular descent and controls spermatogenesis by influencing WNT4 signaling. Development 148, dev191189. 10.1242/dev.191189 33441379 PMC7823160

[B11] Jorgez C. J. Wilken N. Addai J. B. Newberg J. Vangapandu H. V. Pastuszak A. W. (2015). Genomic and genetic variation in E2F transcription factor-1 in men with nonobstructive azoospermia. Fertil. Steril. 103, 44–52. 10.1016/j.fertnstert.2014.09.021 25439843 PMC4282601

[B12] Kaselas C. Florou M. Tirta M. Bitzika S. Sidiropoulou D. Spyridakis I. (2024). The time of diagnosis and surgical treatment of congenital cryptorchidism: a single center's observational study in Greece. Cureus 16, e51580. 10.7759/cureus.51580 38313896 PMC10836180

[B13] Kato T. Mizuno K. Matsumoto D. Nishio H. Nakane A. Kurokawa S. (2022). Low serum inhibin B/Follicle-Stimulating hormones and anti-müllerian hormone/follicle-stimulating hormones ratios as markers of decreased germ cells in infants with bilateral cryptorchidism. J. Urol. 207, 701–709. 10.1097/JU.0000000000002344 34823367 PMC12721657

[B14] Kubarsepp V. Varik K. Varendi H. Antson A. Veinla M. Nellis G. (2022). Prevalence of congenital cryptorchidism in Estonia. Andrology 10, 303–309. 10.1111/andr.13121 34699126

[B15] Kumar A. V. Mills J. Lapierre L. R. (2022). Selective autophagy receptor p62/SQSTM1, a pivotal player in stress and aging. Front. Cell Dev. Biol. 10, 793328. 10.3389/fcell.2022.793328 35237597 PMC8883344

[B16] Lee P. A. Houk C. P. (2013). Cryptorchidism. Curr. Opin. Endocrinol. Diabetes Obes. 20, 210–216. 10.1097/MED.0b013e32835ffc7d 23493040

[B17] Liu J. Xiu W. Sui B. Jin Z. Xu X. Xia N. (2022). Open controversies on the treatment of undescended testis: an update. Front. Pediatr. 10, 874995. 10.3389/fped.2022.874995 35967583 PMC9363670

[B18] Liu S. Yao S. Yang H. Liu S. Wang Y. (2023). Autophagy: regulator of cell death. Cell Death Dis. 14, 648. 10.1038/s41419-023-06154-8 37794028 PMC10551038

[B19] Ma Y. Kurtyka C. A. Boyapalle S. Sung S. S. Lawrence H. Guida W. (2008). A small-molecule E2F inhibitor blocks growth in a melanoma culture model. Cancer Res. 68, 6292–6299. 10.1158/0008-5472.CAN-08-0121 18676853 PMC3615411

[B20] O'Grady S. M. (2019). Oxidative stress, autophagy and airway ion transport. Am. J. Physiol. Cell Physiol. 316, C16–C32. 10.1152/ajpcell.00341.2018 30303690 PMC6383147

[B21] Okugi K. Kuwahara N. Yanome N. Yamada K. Ito T. Takano A. (2022). An *in vitro* system for experimentally induced cryptorchidism. Histochem Cell Biol. 157, 297–307. 10.1007/s00418-022-02078-0 35190876

[B22] Polager S. Ofir M. Ginsberg D. (2008). E2F1 regulates autophagy and the transcription of autophagy genes. Oncogene 27, 4860–4864. 10.1038/onc.2008.117 18408756

[B23] Rew K. T. Langan R. C. Hadj-Moussa M. Heidelbaugh J. J. (2021). Men's health: scrotal and testicular conditions. FP Essent. 503, 23–27. 33856180

[B24] Rodprasert W. Virtanen H. E. Toppari J. (2024). Cryptorchidism and puberty. Front. Endocrinol. (Lausanne) 15, 1347435. 10.3389/fendo.2024.1347435 38532895 PMC10963523

[B25] Shahat A. M. Rizzoto G. Kastelic J. P. (2020). Amelioration of heat stress-induced damage to testes and sperm quality. Theriogenology 158, 84–96. 10.1016/j.theriogenology.2020.08.034 32947064

[B33] Szklarczyk D. Kirsch R. Koutrouli M. Nastou K. Mehryary F. Hachilif R. (2023). The STRING database in 2023: Protein-protein association networks and functional enrichment analyses for any sequenced genome of interest. Nucleic Acids Res. 51 (D1), 638–D646. 10.1093/nar/gkac1000 PMC9825434 36370105

[B26] Thorup J. Hildorf S. Hildorf A. E. Baastrup J. M. Mamsen L. S. Andersen C. Y. (2023). The fate of germ cells in cryptorchid testis. Front. Endocrinol. (Lausanne) 14, 1305428. 10.3389/fendo.2023.1305428 38234428 PMC10792029

[B27] Xiao Y. Cai W. (2020). Autophagy and bacterial infection. Adv. Exp. Med. Biol. 1207, 413–423. 10.1007/978-981-15-4272-5_29 32671764

[B28] Yang M. Yang B. Deng D. (2021). Targeting of EIF4EBP1 by miR-99a-3p affects the functions of B lymphocytes via autophagy and aggravates SLE disease progression. J. Cell Mol. Med. 25, 10291–10305. 10.1111/jcmm.16991 34668631 PMC8572797

[B29] Yefimova M. G. Buschiazzo A. Burel A. Lavault M. T. Pimentel C. Jouve G. (2019). Autophagy is increased in cryptorchid testis resulting in abnormal spermatozoa. Asian J. Androl. 21, 570–576. 10.4103/aja.aja_12_19 31031333 PMC6859671

[B30] Yi D. Liu B. Ding H. Li S. Li R. Pan J. (2023). E2F1 mediates SOX17 deficiency-induced pulmonary hypertension. Hypertension 80, 2357–2371. 10.1161/HYPERTENSIONAHA.123.21241 37737027 PMC10591929

[B31] Zheng Y. Zhang P. Zhang C. Zeng W. (2019). Surgery-induced cryptorchidism induces apoptosis and autophagy of spermatogenic cells in mice. Zygote 27, 101–110. 10.1017/S096719941900011X 30888311

