# Supplementary figures and images for: The role of E2F1 in promoting EIF4EBP1 transcription in cryptorchid mice: association with autophagy in germ cells
Source: Front Genet. 2025 May 23;16:1536672. doi: 10.3389/fgene.2025.1536672 (PMC12141231; doi:10.3389/fgene.2025.1536672)

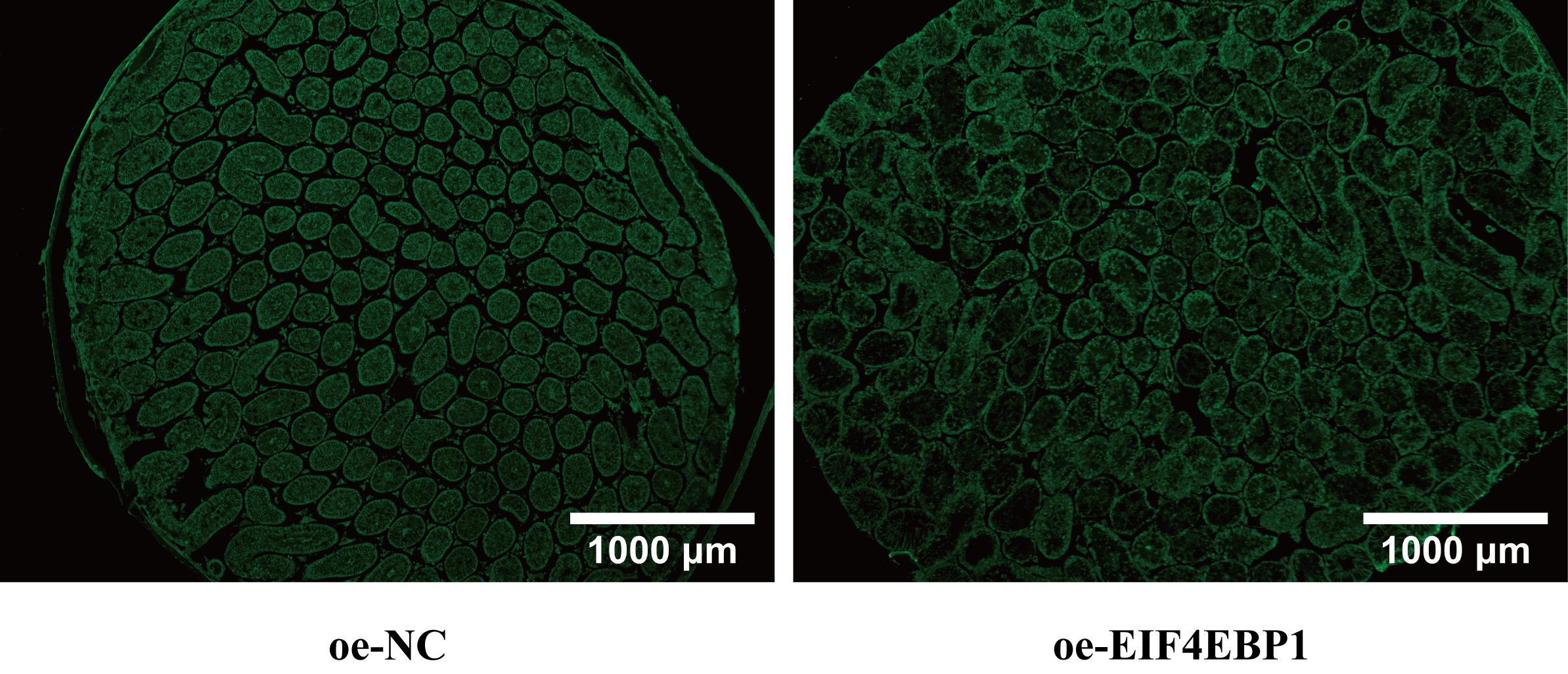

Supplement: Supplementary file 1 [file Image1.tif]
